# A Case Report on Care-Seeking Type Illness Anxiety Disorder after COVID-19 Infection

**DOI:** 10.1155/2023/3003499

**Published:** 2023-12-21

**Authors:** Lakshmi S. Kasi, Bini Moorthy

**Affiliations:** ^1^University of Missouri-Kansas City School of Medicine, Kansas City, MO, USA; ^2^University Health, Kansas City, MO, USA

## Abstract

This case report highlights the diagnostic challenges presented by the overlapping symptoms of illness anxiety disorder (IAD) and long COVID-19 (LC-19). This case report focuses on a 58-year-old woman with care-seeking type IAD in the context of LC-19-associated symptoms. The patient experienced mild COVID-19 in August 2021. Since then, she has reported an increase in LC-19-associated symptoms, including cognitive deficits, breathlessness, fatigue, and anosmia. Despite largely normal laboratory results, imaging, and physical examinations, the patient's distress and care-seeking behaviors persisted, resulting in the diagnosis of IAD. Accurately differentiating between LC-19 and IAD is crucial for appropriate patient care. We discuss the importance of recognizing and treating IAD in patients with LC-19-associated symptoms and the need for further research on the correlation between IAD and both COVID-19 and LC-19.

## 1. Introduction

Illness anxiety disorder (IAD) and long COVID-19 (LC-19) are two distinct conditions that can present with overlapping symptoms and have similarities in presentation. IAD, previously referred to as hypochondriasis, is a psychiatric condition characterized by a preoccupation with having or acquiring a serious illness [1]. Individuals with IAD experience severe anxiety about their health and exhibit excessive health-related behaviors, despite often having normal physical examination and laboratory results [1]. The estimated prevalence of IAD in the general population is about 0.1% [1]. Most individuals with IAD have one of two subtypes: care-seeking type or care-avoidant type [1].

The distinction between care-seeking type IAD and care-avoidant type IAD lies in how individuals respond to their health concerns. Individuals with care-seeking type IAD tend to actively seek medical care and ask to undergo numerous medical tests and treatments [1]. Conversely, individuals with care-avoidant IAD tend to avoid medical care and medical tests despite severe health-related anxiety [1].

Another interesting medical condition is LC-19, which is a novel sequela of COVID-19. While a clear consensus is yet to be reached regarding LC-19, in literature, it has been described to persist for a minimum of 4 weeks after the onset of COVID-19 [2]. It can be continuous, relapsing, or remitting [3]. Symptoms include fatigue, breathlessness, cough, chest tightness, palpitations, myalgia, and neurological and cognitive deficits [2, 3]. Over 40% of adults with a history of COVID-19 are thought to develop LC-19 [4].

We present a 58-year-old woman with care-seeking type IAD in the context of LC-19-associated symptoms. She has care-seeking type IAD as opposed to care-avoidant type IAD since she frequently sought medical care. She regularly saw several physicians and underwent extensive medical work-up. Some LC-19-associated symptoms she experienced include cognitive deficits, breathlessness, fatigue, and anosmia.

## 2. Case Report

### 2.1. Pre-COVID-19 History and Symptoms

The patient is a 58-year-old woman with a psychiatric history of generalized anxiety disorder (GAD), major depressive disorder (MDD), post-traumatic stress disorder (PTSD), dyssomnia, tobacco use disorder, and alcohol use disorder in remission. Her tobacco use disorder began at age 22, and she smoked two packs of cigarettes daily. At age 46, she decreased her tobacco use to smoking one and a half packs of cigarettes daily. Two years later, she further decreased her tobacco use to smoking one pack of cigarettes daily.

Her alcohol use disorder began at age 38, and she drank a pint (473 mL) of hard liquor daily. At age 45, she decreased her alcohol use to half a pint (236.5 mL) of hard liquor daily. The following year, she decreased her alcohol use to only weekends. Two years later, she used alcohol very occasionally. At age 49, her husband passed, and her alcohol use increased to a fifth (750 mL) of gin daily. She was hospitalized twice that year for chronic pancreatitis and quit drinking after the second hospitalization.

She struggled with worsening GAD, MDD, and PTSD since age 39. At age 46, she was prescribed citalopram but did not tolerate it. At age 49, she began regularly seeing outpatient psychiatry. She was prescribed sertraline and prazosin. A few months later, her MDD and PTSD symptoms significantly improved. However, her GAD worsened, and she had more frequent exacerbations. Her sertraline and prazosin dosages were increased. She was also prescribed hydroxyzine but did not tolerate it. Additionally, she received grief therapy as recommended. Her MDD and PTSD symptoms improved significantly more, but her GAD continued to be uncontrolled. Intermittently, she would be out of her medications, and her MDD and GAD would worsen. Her PTSD was stable, even when out of medication.

Also, the patient was prescribed melatonin for her dyssomnia at age 50. Her dyssomnia was uncontrolled despite increasing the melatonin dosage. She did not tolerate trazodone. The patient was then lost to follow-up with outpatient psychiatry for 2 years and returned at age 53. Since then, she has had regular follow-up appointments with outpatient psychiatry.

When she returned to outpatient psychiatry at age 53, she had been off of all psychiatric medications. Her MDD was partially stable. Her PTSD was stable. Her GAD and dyssomnia were uncontrolled. She had resumed drinking small amounts of alcohol on occasion, about once a month, and she continued to smoke one pack of cigarettes daily. Her sertraline was resumed, and she was started on zolpidem. Her dyssomnia improved with zolpidem. Eventually, her sertraline was switched to paroxetine. After trialing multiple medications, she was started on diazepam for a short period for her uncontrolled anxiety. Psychotherapy was also recommended, but she did not take part in it. In April 2020, at age 55, her outpatient psychiatry appointments were converted from in-person to telehealth due to COVID-19 health regulations.

At age 49, the patient first noted fatigue and poor memory. At a follow-up appointment a few months later, she continued to report poor memory. A year later, she reported worsening memory, resulting in losing her keys and forgetting important dates. After that, she did not report fatigue or memory concerns for several years.

### 2.2. Post-COVID-19 History and Symptoms

In August 2021, at age 56, the patient had mild COVID-19, which did not require hospitalization. Since then, for a period of over 1.5 years, she has reported persistent symptoms associated with LC-19. At her following appointment in December 2021, she noted fatigue, breathlessness, ageusia, poor appetite, weight loss, and “not feeling right.” In January 2022, she also reported poor memory, increased confusion, and being easily distracted. She was concerned she had an unnoticed stroke that was causing these symptoms. In February 2022, these symptoms persisted, and she reported left-side weakness. In April 2022, she also reported anosmia, “brain fog,” and feeling disorganized. She stated she could not describe her cognitive symptoms with words. In August 2022, she also reported generalized weakness, loss of balance, dizziness, body temperature changes, and increased disorganization. She stated she couldn't go to walk-in psychotherapy due to her disorganization.

At this time, she also began experiencing significant impairments in her daily functioning. She reported difficulties with instrumental activities of daily living, such as cooking. In September 2022, she also reported trouble with word finding. In October 2022, she also reported concern regarding her sensation of touch and vision. She reported that things felt different, and she thought her vision “easily missed things.” For example, she would not have initially seen a trash bag and would suddenly notice it was there. In January 2023, she also expressed feeling unable to drive, although once she was in the car, she could complete the task. In March 2023, she noted not being able to feel her left hand and believed she may have had a stroke to cause this. Other than fatigue and difficulty with memory, the patient did not express any of these symptoms or concerns prior to August 2021.

Her social isolation, due to her symptoms, further exacerbated her distress. She reported excessive anxiety regarding the uncertain etiology of her symptoms. She frequently expressed concern that her physicians were not doing enough to diagnose and treat her. She requested a comprehensive work-up and hospital admission. She did not meet the criteria for hospital admission and was not admitted.

### 2.3. Assessment

Despite the patient's alcohol use disorder in remission, no organic basis for cognitive impairment was revealed over the years. Since age 49, she has seen outpatient neurology several times. At age 50, neurological examinations revealed decreased strength bilaterally in distal upper extremities (4/5), decreased strength bilaterally in proximal lower extremities (4/5), and decreased strength bilaterally in distal lower extremities (3/5). The patient had sensory loss on a stocking distribution in bilateral lower extremities, including pinprick, temperature, touch, and vibration. Ankle reflexes were also absent bilaterally. These findings have been unchanged over the years. No other neurological abnormalities were revealed on physical examinations.

At age 50, she also had lab work done to assess for an underlying condition. Her vitamin B12, folate, homocysteine, antinuclear antibody (ANA), sedimentation rate, Lyme panel, thyroid stimulating hormone (TSH), and serum protein electrophoresis were all within normal limits. She had low thiamine (7 nmol/L), low methylmalonic acid (69 nmol/L), elevated vitamin E (beta gamma tocopherol 4.6 mg/L), and slightly elevated hemoglobin A1c (5.7%), which were appropriately treated. In addition, she has had five electromyography tests at ages 49, 51, 52, 53, and 55, which were consistent with axonal sensory peripheral neuropathy. This is thought to be secondary to her alcohol use. She had a brain MRI at age 41, which revealed a nonspecific tiny focal area of abnormal signal within the right periarterial region of the lateral ventricle. Early mild small vessel ischemic disease and early demyelinating process could not be excluded. Brain MRI at age 50 revealed no acute intracranial process, mass effect, or infarct. It also revealed a well-circumscribed 6 mm T1 hyperintense focus along the dorsal pituitary. However, the pituitary was normal size, and there was no mass effect on the optic chiasm or midline shift of the infundibulum.

After having COVID-19 at age 56, she had additional work-up. However, no organic basis for cognitive impairment and her other symptoms were revealed. Out-patient laboratory tests revealed low folate (2.8 mg/mL) and low vitamin B1 (7 nmol/L), possibly secondary to her alcohol use. These were appropriately treated. Basic metabolic panel, complete blood count, glomerular filtration rate, calcium, magnesium, phosphorus, aspartate aminotransferase, alanine aminotransferase, TSH, vitamin B6, and vitamin B12 were largely within normal limits. ANA screen, centromere antibody, RNA antibody, smith antibody, DNA double-stranded antibody, Sjogren's antibody, Jo-1 antibody, scleroderma antibody, chromatin, ribosomal p, and snRNP were all negative. Urinalysis was also negative. A repeat brain MRI in February 2022, at age 57, revealed no acute infarct, hemorrhage, intracranial mass, or abnormal enhancement. The Saint Louis University Mental Status Examination also yielded normal findings. She scored 22/30 (*n* ≥ 26) on the Montreal Cognitive Assessment, which indicated mild cognitive impairment but did not fully explain her symptoms. She was disappointed that she did not score lower since she thought that would have explained her symptoms. Apart from her anxiety, distress, and known neurological abnormalities, her physical and mental status examinations were unremarkable.

### 2.4. Treatment and Outcome

Despite the absence of objective measures to explain her symptoms, the patient's distress and care-seeking behaviors persisted. The patient was treated with a multidisciplinary approach. The patient's primary care physician (PCP) recommended corticosteroid sinus lavage and irrigation for her anosmia and ageusia, which the patient declined. She sought care with a neurologist for her symptoms, who suspected a psychosomatic component to her symptoms. She also sought care with a pulmonologist for a pulmonary nodule and emphysema during this period. Throughout the 1.5 years following her initial COVID-19 diagnosis, the patient received regular outpatient psychiatric care, which culminated in a diagnosis of care-seeking type IAD.

The patient declined psychotherapy for IAD, which is first-line treatment [1]. She had poor adherence to medications, which included a multimodal approach with sertraline, zolpidem, clonazepam, pregabalin, and vitamin supplements. She experienced partial relief in her LC-19-associated symptoms, anxiety, and distress with these psychiatric medications. However, she continues to report moderate symptoms and impairments in her daily life. At this time, she continues to receive ongoing outpatient psychiatric care to address her IAD, among other psychiatric conditions. She is also regularly seen by her PCP.

## 3. Discussion

### 3.1. Distinguishing between LC-19 and IAD

The patient has care-seeking type IAD in the setting of LC-19-associated symptoms after experiencing mild COVID-19. LC-19 remains poorly defined due to the wide variation in symptom presentation, symptom timeline, and nonspecific nature of LC-19 symptoms [5]. In literature, LC-19 has been described to persist for a minimum of 4 weeks after the onset of COVID-19 [2]. Symptoms can last from weeks to months [6] and can be continuous, relapsing, or remitting [3]. The most common symptoms of LC-19 are breathlessness, fatigue, anosmia, and myalgia [7]. The patient had all of these other than myalgia. Other LC-19 symptoms the patient reported include problems with concentration, “brain fog,” loss of appetite, and ageusia [7]. However, the patient also had many other symptoms, including changes in vision, inability to cook, and feeling unable to drive, which have not been described in other cases of LC-19. It is also important to note that patients who experienced severe COVID-19 illness, especially those who were hospitalized or needed intensive care, are at increased risk for LC-19 compared to patients who experienced mild COVID-19 [8], like this patient. While the patient has some characteristics of LC-19, some aspects of her presentation are inconsistent with LC-19.

In contrast, IAD has much clearer diagnostic criteria [1], which the patient more definitively meets. The patient demonstrated care-seeking behavior and excessive preoccupation with her health for greater than 6 months despite mostly normal laboratory findings, imaging, and physical examinations. This is consistent with IAD. Furthermore, the patient's symptoms have significantly disrupted her daily functioning, which is also commonly seen in IAD [1]. While the cause of IAD remains unknown, a major risk factor for developing IAD is having an underlying anxiety disorder such as GAD or PTSD [9], both of which the patient had.

### 3.2. Treatment Approaches

Distinguishing between LC-19 and IAD is essential to appropriately treat patients. While there is no specific treatment for LC-19, managing associated symptoms and addressing its various components require a multidisciplinary approach. This includes referrals to cardiologists, neurologists, pulmonologists, rheumatologists, and other specialists [10]. On the other hand, treatment for IAD includes psychotherapy, regular appointments with a PCP, and regular appointments with a psychiatrist [1]. A primary goal of treating patients with known IAD is to avoid unnecessary imaging, lab work, and referrals to specialists [1]. Accurately diagnosing IAD and distinguishing it from LC-19 is essential for appropriate patient care, avoiding unnecessary medical tests, and optimizing overall treatment. Identifying the presence of a coexisting medical condition in a patient with known IAD can be challenging.

### 3.3. Future Research Directions

Of note, the prevalence of anxiety has increased worldwide by 25% since COVID-19 [11]. The correlation between IAD and COVID-19 remains unexplored in the United States on a large scale. A cross-sectional study conducted in Iran found the prevalence of IAD in individuals whose relatives had a history of COVID-19 was 5.32 times higher than the prevalence of IAD in the general population [12]. The correlation between IAD and, specifically, LC-19 has not been published, yet. Future research may wish to investigate the change in prevalence of IAD in the general population since COVID-19. Future research may also wish to investigate the change in the prevalence of IAD in patients with a history of COVID-19 and in patients with a history of LC-19. We hypothesize there has been an increase in the prevalence of IAD in all of these instances. Hence, it is very important for clinicians to accurately diagnose patients with IAD and appropriately treat these patients.

## 4. Conclusion

In conclusion, this case report emphasizes the importance of recognizing and treating IAD in patients with LC-19-associated symptoms. Accurate diagnosis is vital to optimize patient care, avoid unnecessary medical tests, and avoid unnecessary appointments with specialists. Furthermore, future research is needed to better understand the association between IAD and both COVID-19 and LC-19, which would provide valuable insights into potential correlations and prevalence rates.

## Data Availability

No underlined data were used/generated during the study.
